# Anandamide-ceramide interactions in a membrane environment: Molecular dynamic simulations data

**DOI:** 10.1016/j.dib.2017.07.024

**Published:** 2017-07-14

**Authors:** Coralie Di Scala, Morgane Mazzarino, Nouara Yahi, Karine Varini, Nicolas Garmy, Jacques Fantini, Henri Chahinian

**Affiliations:** a Faculté des Sciences, Aix-Marseille Université, EA4674 Marseille, France; b Aix-Marseille Université, CNRS UMR7259, Marseille, France

## Abstract

Anandamide is a lipid neurotransmitter that interacts with various plasma membrane lipids. The data here consists of molecular dynamics simulations of anandamide, C18-ceramide and cholesterol performed in vacuo and within a hydrated palmitoyl-oleoyl-phosphatidylcholine (POPC)/cholesterol membrane. Several models of anandamide/cholesterol and anandamide/ceramide complexes are presented. The energy of interaction and the nature of the intermolecular forces involved in each of these complexes are detailed. The impact of water molecules hydrating the POPC/cholesterol membrane for the stability of the anandamide/cholesterol and anandamide/ceramide complexes is also analyzed. From a total number of 1920 water molecules stochatiscally merged with the lipid matrix, 48 were eventually redistributed around the polar head groups of the anandamide/ceramide complex, whereas only 15 reached with the anandamide/cholesterol complex. The interpretation of this dataset is presented in the accompanying article “Ceramide binding to anandamide increases its half-life and potentiates its cytotoxicity in human neuroblastoma cells” [1].

**Specifications Table**

**Table:** 

Subject area	*Biology*
More specific subject area	*Molecular neurosciences*
Type of data	*Text file and figures*
How data was acquired	*Computer, Hyperchem program*
Data format	*Snapshots of molecular dynamics simulations*
Experimental factors	*Geometry optimization of each molecule with the Polak-Ribiere algorithm*
Experimental features	*Iterative rounds of molecular dynamics simulations with the CHARMM force field*
Data source location	*Not applicable*
Data accessibility	*All data are presented in this article*

**Value of the data**

• The data provides a series of molecular models of anandamide interacting with cholesterol and/or ceramide.
• The calculation of the energy of interaction of anandamide/ceramide and anandamide/cholesterol complexes gives an estimation of the relative affinity of anandamide for each lipid in the membrane environment.
• The calculations in presence of water molecules hydrating the membrane are important for evaluating the impact of lipid head groups hydration on the stability of each anandamide/lipid complex.
• Researchers interested in investigating the neurotransmitter activity of anandamide might be aware that anandamide can select and interact with distinct membrane lipids in the plasma membrane of neural cells.

## 1 Data

Anandamide (AEA) is a lipid neurotransmitter derived from arachidonic acid that inserts in the plasma membrane of target cells prior to exerting its biological effects [2]. Therefore, the way AEA interacts with membrane lipids is of critical importance to understand its mechanism of action at the molecular level. Previous data showed that AEA interacts with cholesterol and uses this lipid as an efficient transport shuttle to cross the plasma membrane [3]. The dataset presented here contains a series of snapshots of molecular dynamics studies of AEA interacting with either cholesterol or ceramide in a palmitoyl-oleoyl-phosphatidylcholine membrane containing dispersed cholesterol molecules (typical Ld phase [4], [5]) and hydrated by several layers of water molecules.

## 2 Experimental design, materials and methods

### 2.1 Molecular modeling simulations

Geometry optimization of each cholesterol/AEA and C18-ceramide/AEA complexes was first achieved with Polak-Ribiere algorithm [6]. The complexes were then merged within a phosphatidylcholine (POPC) matrix containing cholesterol molecules (molecular ratio POPC:cholesterol 10:1) as previously described [7]. Molecular dynamics simulations with the CHARMM force field were performed in vacuo or in presence of several layers of water molecules with the Hyperchem program (ChemCAD, Obernay, France) [8], [9]. A step-by-step description of these molecular modeling approaches has been published by Di Scala and Fantini [9]. Further interesting insight in the modeling approach for membrane lipids can be found in the literature [10], [11], [12].

### 2.2 Molecular modeling of the AEA/C18-ceramide and AEA/cholesterol complexes in vacuo

**Fig. 1:**
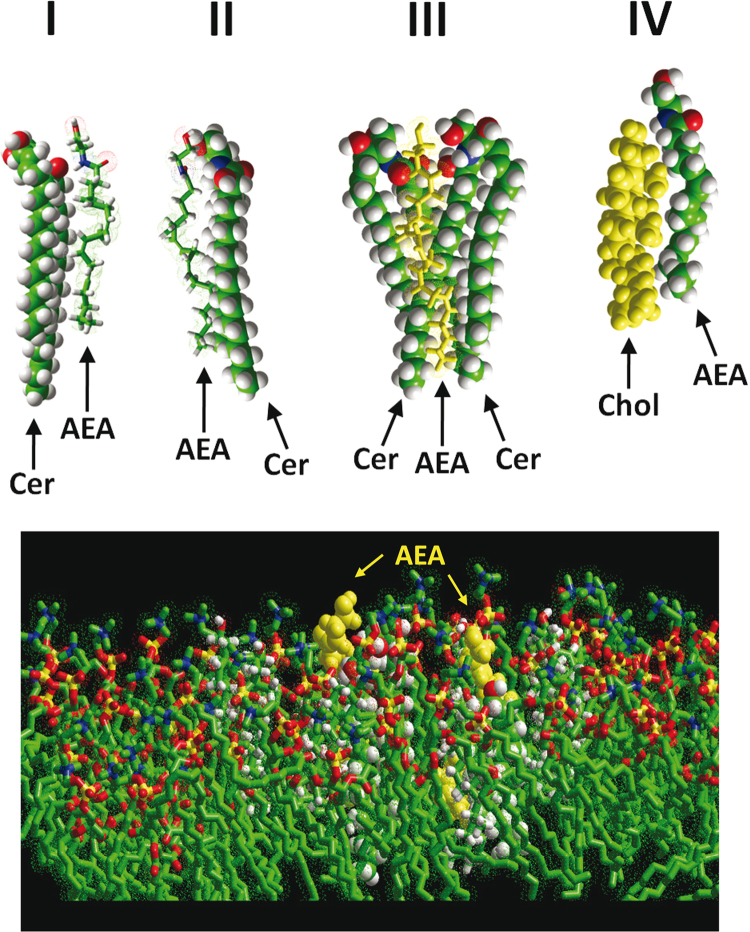
Molecular dynamics simulation of AEA-lipid interactions in vacuo.**Note:** The upper panel shows the introduction of AEA and ceramide or cholesterol in a phosphatidylcholine (POPC)/cholesterol matrix. After geometry optimization of the system, molecular dynamics simulations were conducted for iterative periods of 1 ns, until a stable complex was formed (total time 10 ns). Snapshots of AEA/ceramide complexes (models I, II and III) and AEA/cholesterol (model IV) are shown (the POPC/cholesterol matrix is not represented to improve clarity). Models I and II show two ways to construct a binary complex between AEA and ceramide. Models I and II are not mutually exclusive. Model III corresponds to a combination of models I and II with AEA bound to two ceramide molecules. Model IV shows a complex between AEA and cholesterol [3], to be compared with the models of AEA/ceramide complexes. The lower panel represents AEA/ceramide (AEA in yellow spheres on the right) and AEA/cholesterol (AEA in yellow spheres on the left) inserted in POPC/cholesterol bilayer. It is interesting to note the difference in the degree of AEA insertion relatively to its partner (ceramide or cholesterol). The position of the ethanolamine moiety is freely accessible to water in the case of the AEA/cholesterol complex, and more deeply embedded in the membrane in the case of AEA/ceramide complex. One can see that cholesterol lets the amide group of AEA accessible for fatty acid amide hydrolase (FAAH) hydrolysis, whereas ceramide does not. These observations could be respectively correlated with the cholesterol-induced interfacial activation of FAAH and the ceramide-inhibition of FAAH.

Molecular modeling simulations have been first carried out in vacuo to assess if a stable AEA/ceramide complex could be formed within a POPC/cholesterol membrane (starting conditions consisting of 120 POPC molecules stochastically merged with 12 cholesterol, for a molar ratio of 10 POPC per cholesterol). The respective conformations of AEA and ceramide in the membrane environment are consistent with the formation of two types of binary AEA/ceramide complexes (models I and II) which are not mutually exclusive and can even be associated in a ternary complex (model III) constituted by one AEA molecule interacting with two ceramide molecules (Fig. 1). The AEA/ceramide complex (model I) is stabilized by numerous van der Waals interactions and a hydrogen bond involving the carbonyl group of ceramide and the NH group of AEA at a distance of 2.3 Å. The alternative AEA/ceramide complex (model II) is also stabilized by numerous van der Waals interactions and a hydrogen bound involving the carbonyl group of AEA and the NH group of ceramide at a distance of 2.9 Å. Overall, the energy of interaction of these AEA/ceramide complexes was estimated to -43.0 kJ mol^-1^ (model I) and -47.7 kJ mol^-1^ (model II). In the ternary complex (model III), the two hydrogen bounds described in models I and II coexist, yet with interatomic distances slightly modified (2.0 Å and 2.8 Å). The energy of interaction of the ternary complex was -84.6 kJ mol^-1^, a value close to the sum of the energy of interaction of each binary complex (-90.7 kJ mol^-1^). For comparison, the energy of interaction of the AEA/cholesterol complex (model IV) was significantly lower (-30.3 kJ mol^-1^) [3]. The conformation of cholesterol in this complex is consistent with previously characterized cholesterol conformers in membrane environment [9].

### 2.3 Molecular modeling of the AEA/C18-ceramide and AEA/cholesterol complexes within a hydrated membrane

**Fig. 2:**
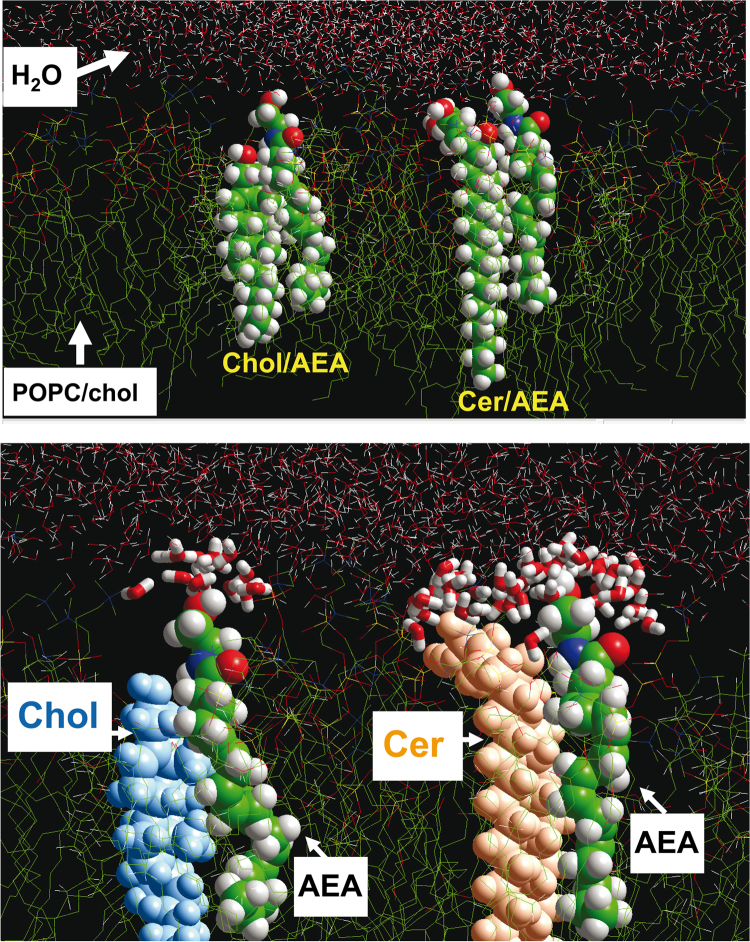
Molecular dynamics simulation of AEA-lipid interactions within a fully hydrated POPC/cholesterol membrane.**Note:** The upper panel shows a snapshot of the POPC/cholesterol matrix hydrated with 1920 molecules of water and merged with AEA/cholesterol and AEA/ceramide complexes. After an initial step of geometry optimization of the whole system, two rounds of iterative simulation periods of 1 ns (total time 2 ns) were conducted. All molecules are represented in sticks whereas the AEA/cholesterol and AEA/ceramide complexes are in spheres rendition. The lower panel shows a snapshot taken after 10 ns of simulation. The water molecules hydrating the polar head groups of the AEA/cholesterol and AEA/ceramide complexes are in tubes representation. Cholesterol is in blue, ceramide in orange, and anandamide in atoms colors (carbon in green, oxygen in red, nitrogen in blue, and hydrogen in white).

In a second series of experiments, we have performed similar rounds of molecular dynamics simulations in presence of several layers of water molecules (initial conditions with 1920 molecules of water randomly distributed above the polar head groups of the lipid matrix). Two snapshots of these simulations are presented in Fig. 2A (after 2 ns) and 2B (after 10 ns). The presence of water molecules hydrating the lipid membrane reinforced the association of AEA with both cholesterol and ceramide. Indeed, the AEA/ceramide complex was hydrated with 48 molecules of water which appeared to be homogeneously distributed around the polar head groups of both lipids (Fig. 2B). In contrast, the hydration shell of the AEA/cholesterol complex involved only 15 water molecules, all interacting with the terminal OH group of AEA. The difference of the hydration level of both complexes is especially visible in Fig. 2B with the concerned water molecules in tube rendering. Apart from these selective hydration properties, the AEA/ceramide and AEA/cholesterol complexes that were simulated within a fully hydrated POPC/cholesterol membrane displayed energy interaction values similar to those obtained in vacuo.
